# Dry Period Heat Stress Impacts Mammary Protein Metabolism in the Subsequent Lactation

**DOI:** 10.3390/ani11092676

**Published:** 2021-09-13

**Authors:** Bethany Dado-Senn, Amy L. Skibiel, Geoffrey E. Dahl, Sebastian I. Arriola Apelo, Jimena Laporta

**Affiliations:** 1 Department of Animal and Dairy Sciences, University of Wisconsin-Madison, Madison, WI 53706, USA; bethany.senn@wisc.edu (B.D.-S.); arriolaapelo@wisc.edu (S.I.A.A.); 2 Department of Animal, Veterinary and Food Sciences, University of Idaho, Moscow, ID 83844, USA; askibiel@uidaho.edu; 3 Department of Animal Sciences, University of Florida, Gainesville, FL 32608, USA; gdahl@ufl.edu

**Keywords:** hyperthermia, milk, late-gestation

## Abstract

**Simple Summary:**

Heat stress during the dry period of dairy cows reduces milk yield in the following lactation. Factors such as altered mammary metabolism could impact yields and alter milk composition, including milk protein. We sought to determine if exposure to dry period heat stress would influence mammary milk protein metabolism during the subsequent lactation. Objectives were to first determine the impact of dry period heat stress on milk protein yields and secondly characterize the amino acid and protein profiles in the mammary tissue, milk, and blood to elucidate potential carry-over impact of dry period heat stress on systems that participate directly in milk protein metabolism (i.e., mTOR). We found that heat stress during the dry period reduces milk yield, protein content, and protein yield in the subsequent lactation. The plasma amino acid profile and mammary amino acid transporters are altered in dry period heat-stressed cows, and mammary mTOR signaling proteins are differentially expressed as well. It appears that dry period heat stress impacts mammary metabolism with consequences on milk yield and protein content. The continuous production of high-quality and -quantity milk is vital as a sustainable source of protein in the face of rising global temperatures.

**Abstract:**

Dry period heat stress impairs subsequent milk production, but its impact on milk protein content and yield is inconsistent. We hypothesize that dairy cow exposure to dry period heat stress will reduce milk protein synthesis in the next lactation, potentially through modified amino acid (AA) transport and compromised mTOR signaling in the mammary gland. Cows were enrolled into heat-stressed (dry-HT, *n* = 12) or cooled (dry-CL, *n* = 12) treatments for a 46-day dry period then cooled after calving. Milk yield and composition and dry matter intake were recorded, and milk, blood, and mammary tissue samples were collected at 14, 42, and 84 days in milk (DIM) to determine free AA concentrations, milk protein fractions, and mammary AA transporter and mTOR pathway gene and protein expression. Dry matter intake did not significantly differ between treatments pre- or postpartum. Compared with dry-CL cows, milk yield was decreased (32.3 vs. 37.7 ± 1.6 kg/day) and milk protein yield and content were reduced in dry-HT cows by 0.18 kg/day and 0.1%. Further, dry-HT cows had higher plasma concentrations of glutamic acid, phenylalanine, and taurine. Gene expression of key AA transporters was upregulated at 14 and 42 DIM in dry-HT cows. Despite minor changes in mTOR pathway gene expression, the protein 4E-BP1 was upregulated in dry-HT cows at 42 DIM whereas Akt and p70 S6K1 were downregulated. These results indicate major mammary metabolic adaptations during lactation after prior exposure to dry period heat stress.

## 1. Introduction

High ambient temperatures and relative humidity negatively impact dairy production, costing the U.S. dairy industry nearly $1 billion annually due to lower milk yield of lactating cows alone [1]. Physiological heat stress occurs when elevated ambient temperature and humidity push an animal past the upper critical temperature (UCT) limit of the thermoneutral zone. To acclimate, animals adapt physiology and behavior to reduce heat production and increase heat loss [2]. Lactating dairy cattle are particularly susceptible to hyperthermia due to high metabolic rates and production demand. Heat stress responses are initiated above skin-surface temperature of 35 °C or a temperature-humidity index (THI) as low as 68 [3,4,5]. Initial behavioral and physiological responses in dairy cattle include reduced feed intake and energy diversion away from production, such as reduction in milk yield or impaired milk component synthesis, including milk protein [6,7,8]. 

Hyperthermia in lactating dairy cows leads to decreased milk protein synthesis beyond the anticipated losses from reduced dry matter intake alone [8,9,10,11]. The reduction in overall milk protein content is attributed in part to several physiological and molecular systems, including a shift in blood flow away from splanchnic tissues and the mammary gland to peripheral tissues [12,13], reduced availability of total and individual amino acids (AA) for milk protein synthesis [9,14,15], and alterations in cell-signaling activity of pathways regulating milk protein synthesis such as the mechanistic target of rapamycin complex 1 (mTORC1) [10,16]. Indeed, in vitro studies of high incubation temperature on bovine mammary epithelial cells (bMEC) indicate a downregulation of genes involved in AA utilization and protein transcription, increased gene expression of AA transporters, and impaired mTORC1 kinase activity, all possibly leading to a reduction in milk protein synthesis [16,17]. Milk protein fractions are also altered under heat stress [11,18].

While dry, non-lactating cows generate less metabolic heat and have a higher UCT to their thermoneutral zone than lactating cows [19], heat stress during the dry period can still negatively impact milk production in the subsequent lactation. Relative to cows cooled with fans and soakers, cows heat-stressed during the dry period produce an average of 3.6 kg/day less milk even when all cows are provided active cooling after calving [20]. Lack of dry cow cooling could cost the dairy industry about $800 million annually due to decreased milk yield in the dam alone [21]. However, this does not account for lost premiums from reduced milk protein percentage, as the impact of dry period heat stress on milk protein fraction and content is unclear. Past studies report conflicting results; cows exposed to dry period heat stress show no difference, slight decreases, or even moderate increases in milk protein content or yield in the next lactation [22,23,24]. 

The objectives of the present study were to measure alterations in milk protein content and yield after exposure to dry period heat stress and to determine any carry-over effects on milk protein synthesis regulation during lactation, specifically AA transport and mTORC1 signaling. We hypothesized that exposure of dry cows to heat stress would reduce milk protein content and yield across the next lactation by impairing the capacity for milk protein synthesis through altered AA availability and reduced mTORC1 signaling.

## 2. Materials and Methods

### 2.1. Animals and Experimental Design

This study occurred between May to December 2016 at the University of Florida Dairy Unit (Hague, FL, USA) with a herd of multiparous Holstein cows as previously described by Dado-Senn et al. 2019 [25]. All procedures were approved by the UF Institutional Animal Care and Use Committee (Protocol #201508730). Cows were dried off at 46 days before expected calving date according to Dairy Unit standard operating procedures and randomly assigned to treatments based on previous lactation mature-equivalent milk production and parity (1.7 ± 0.8 vs. 1.8 ± 1.3 lactations for cooled vs. heat-stressed cows, respectively). The two treatments applied for the duration of the dry period were heat stress (dry-HT; *n* = 12) with access to a shaded, sand-bedded free-stall barn or cooling (dry-CL; *n* = 12) with access to shade plus water soakers and fans. Dry period treatment occurred from May to September in a subtropical climate. Heat stress was considered achieved when the temperature-humidity index (THI) exceeded 68 [4]. Barn ambient temperature and relative humidity were recorded every 15 min across the dry period with Hobo Pro series Temp probes (Onset Computer Corp., Pocasset, MA, USA) to calculate THI [26,27]. The THI during the dry period averaged 76.1 ± 3.7 over the duration of the dry period treatment. During the dry period, cows were fed a standard total mixed ration (TMR), and individual dry matter intake (DMI) was recorded using a Calan gate system. After calving, during the entire lactation all cows were fed a lactating cow TMR and housed as a group in shaded, free-stall barns with free access to water soakers and fans (i.e., actively cooled), where THI averaged 64.4 ± 7.9. Postpartum DMI was measured up to 42 days in milk (DIM). Cows were milked twice daily according to standard operating procedure. 

### 2.2. Physiological Measures and Milk Yield

During the dry period, respiration rate (RR) was measured thrice weekly at 14:00 h by counting flank movements per minute and rectal temperature (RT) was recorded twice daily at 07:30 h and 14:30 h. Post-calving RR and RT were recorded at 14:00 h every 7 days until 84 DIM. Daily milk yield, component concentration (i.e., fat, protein, fat:protein ratio, and lactose), and component yield were obtained from AfiFarm Dairy Herd Management Software using milk meters and near-infrared spectroscopy, respectively (Afimilk Ltd., Kibbutz, Afikim, Israel) up to 210 DIM. The range for accuracy for this system is between 2–6% for fat, 2–5% for protein, and no range given for lactose [28]. Colostrum yield and composition were also measured; colostrum was considered the milk collected at the first milking after calving only (i.e., 0 DIM).

### 2.3. Milk and Blood Profile Analysis

After calving, milk samples (from *n* = 6 cows per treatment) were collected at 14, 42, and 84 DIM at approximately 11:00 h using standard DeLaval milk collection kits. Milk was stored at −20 °C until analysis. Samples were shipped to the University of South Dakota Dairy Manufacturing laboratory to determine milk protein profile percentages using high-performance liquid chromatography according to standard operating procedures of the laboratory [29]. Blood samples (from *n* = 5 cows per treatment) were collected at 14, 42, and 84 DIM at approximately 15:00 h from coccygeal vessels into sodium-heparinized vacutainers (BectonDickinson and Co., Franklin Lakes, NJ, USA). After collection, blood samples were promptly placed on ice and centrifuged at 3000× *g* for 20 min within 1 h after collection. After centrifugation, plasma samples were frozen at −20 °C until analysis. Samples were analyzed by the Experiment Station Chemical Laboratories at the University of Missouri for free amino acid analysis using cation-exchange chromatography (cIEC-HPLC) coupled with post-column ninhydrin derivatization and quantitation [30]. All subsampling was collected from a subset of the *n* = 12 cows per treatment used to measure physiological measures and milk yield. 

### 2.4. Mammary Biopsies 

Mammary biopsies were collected on 14, 42, and 84 DIM from a subset of cows that remained the same across sampling (from *n* = 6 cows per treatment), according to the methods of Farr et al. with modifications previously described in Dado-Senn et al. 2019 [25,31]. After sedation and sterilization, a stainless-steel biopsy tool attached to a drill was inserted into an incision in the mammary gland to cut a core of parenchymal tissue. The tissue was immediately washed in sterile saline, trimmed, and sectioned, and stored in RNAlater at −20 °C or flash frozen and stored at −80 °C. 

### 2.5. RNA Isolation and qRT-PCR

Total RNA was extracted from 49.7 ± 5.9 mg of mammary tissue (*n* = 6 per treatment per time) using the RNeasy Mini Kit (catalog #74104, Qiagen, Valencia, CA, USA) according to the manufacturer’s instructions. RNA concentration was determined on Qubit 2.0 Fluorometer (ThermoFisher, Invitrogen, Grand Island, NY, USA). RNA purity (A260/A280) for all samples averaged 1.9 ± 0.5. A total of 1 μg RNA from each sample was used to synthesize cDNA using the iScript cDNA synthesis kit (Bio-Rad Laboratories, CA, USA) and diluted 1:5 in ddH_2_O. Gene expression was measured by quantitative real-time PCR (qRT-PCR) with the CFX96 Touch Real-Time PCR Detection System (Bio-Rad). Genes selected for analysis included known bovine mammary gland amino acid transporter genes *solute carrier* (*SLC*) *1A1*, *1A5*, *3A2*, *7A1*, *7A5*, and *36A1* and bovine mTOR pathway target genes *unc-51 like autophagy activating kinase 1* (*ULK1*), *ribosomal protein S6 kinase beta-1* (*p70 S6K*), *ribosomal protein S6* (*rpS6*), *protein kinase B* (*Akt*), and *eukaryotic translation initiation factor 4E-binding protein 1* (*4E-BP1*). Reaction mixtures were completed as previously described [32] and cycling conditions were: 1 cycle for 3 min at 95 °C, 50 cycles of 10 s at 95 °C, and 30 s at 60 °C, followed by melt curve measurement from 65 °C to 95 °C in 0.5° increments for 5 s. Positive and negative non-template controls were added to each PCR plate. Each sample was assessed in duplicate and the %CV between the duplicates was <2%. Genes were validated before use (Supplementary Figure S1). Primer sequences for the validated genes were obtained from the literature [33] or specifically designed to span exon-exon junctions to minimize the potential of amplifying genomic DNA using Primer3 software (Supplementary Table S1). Either the geometric mean between three housekeeping genes (*ribosomal protein S9*, *RPS9*, *ubiquitously expressed prefoldin-like chaperone*, *UXT*, and *eukaryotic initiation factor 4F*, *EIF4*; AA transporter genes) or *UXT* alone (mTOR genes), selected based on literature review [34] were used to calculate the relative gene expression using the method 2^-ΔΔCt^, with dry-CL as the reference group [35]. Only *UXT* was used for mTOR genes due to similarity of *RPS9* and *EIF4* to mTOR genes measured. 

### 2.6. Protein Extraction and Western Blotting

Frozen tissues were lysed using a Mini-Beadbeater-24 (BioSpec Products, Bartlesville, OK, USA) and 1 mm glass beads in RIPA buffer containing 50 mM Hepes, 40 mM NaCl, 2 mM EDTA, 1.5 mM Na_3_VO_4_, 50 mM NaF, 10 mM Na_4_P_2_O_7_, and 10 mM C_3_H_7_Na_2_O_6_P, supplemented with Halt™ Proteases and Phosphatases Inhibitor Cocktail (#1861282, Thermo Scientific™, Waltham, MA, USA). Proteins were isolated by centrifugation for 15 min at 18,000× *g*. Lysate protein concentration was determined by bicinchoninic acid assay (#71285, Millipore Sigma, Darmstadt, Germany) and standardized to 1.5 mg/mL in sodium dodecylsulfate (SDS) sample buffer (Laemmli, Bio-Rad #161-0747). Thirty micrograms of protein were denatured at 95 °C for 10 min, and separated by SDS polyacrylamide gel electrophoresis on either 16% or 8% Novex™ Tris-glycine gels (ThermoFisher Scientific, Waltham, MA, USA) for 35 min at 200 V. Proteins were transferred to nitrocellulose membranes (60 min at 20 V) and membranes blocked with Odyssey^®^ Blocking Buffer (LI-COR Biosciences, Lincoln, NE, USA) for 60 min. Proteins were probed overnight against target primary antibodies from Cell Signaling Technology (Danvers, MA, USA): 4E-BP1 (#9644), Actin (#4970), Akt (#4060), and p70 S6K1 (#2708). All primary antibodies were diluted 1:1000 in blocking buffer. Primary antibodies were probed against anti-rabbit or anti-mouse horseradish peroxidase-linked secondary antibodies (Cell Signaling Technology, Danvers, MA, USA) diluted 1:2000 in blocking buffer. Protein signaling was detected in an Odyssey FC imaging system (LI-COR Biosciences, Lincoln, NE, USA) and chemiluminescence signal was quantified in Image Studio software (LI-COR Biosciences, Lincoln, NE, USA). 

### 2.7. Mammary Tissue AA Concentration

For tissue AA concentration, 60.9 ± 27.4 mg of mammary tissue was mixed with PBS and a known amount of universally labeled ^13^C AA mix as an internal standard. The tissue was bead-homogenized, centrifuged at 12× *g* for 10 min, and the supernatant was deproteinized with 0.5 M perchloric acid. Amino acids were derivatized following EZ-Faast kit instructions (CN KH0-7338, Phenomenex, Torrance, CA, USA). The tissue concentration of the derivatized AA was measured in a Shimadzu 2020 liquid chromatography mass spectrometer (Shimadzu, Kyoto, Japan) as previously reported [36]. 

### 2.8. Statistical Analyses

Statistical analysis was conducted in SAS v. 9.4 (SAS Institute, Cary, NC, USA). Continuous data (i.e., physiological measures and milk yield) were analyzed by generalized linear mixed models using PROC MIXED with fixed effects of treatment, time (day or week-in-milk as repeated measures), and their interaction. Data collected at 14, 42, and 84 DIM (i.e., milk and blood profiles and mammary tissues analyses) were analyzed by generalized linear mixed models using PROC MIXED with fixed effects of treatment, time (14, 42, and 84 DIM), and their interaction. Colostrum variables were analyzed in PROC MIXED without repeated measures and ID within treatment was considered random. The covariate analysis used was the first-order autoregressive covariance structure (AR-1). Residuals were tested for normality, and data were log-transformed as needed. Significance was declared at *p* ≤ 0.05 and tendency was declared at 0.05 < *p* ≤ 0.10. *p*-values listed in text are for the main effect of treatment (i.e., TRT) unless otherwise stated. Data are presented as least squares means (LSM) ± standard error (SE) unless otherwise stated. 

## 3. Results

### 3.1. Physiological Measures and Milk Yield

Results related to environment, DMI, and vital responses were previously reported [25,37]. Briefly, dry period (i.e., prepartum) DMI was not statistically different between treatments (9.8 vs. 11.9 ± 1.2 kg/day for dry-HT vs. dry-CL, respectively, *p* = 0.24), but RR and RT were higher in dry-HT relative to dry-CL cows (*p* < 0.01, and *p* < 0.01, respectively). This indicates that dry period heat stress abatement in the cooled group was successful in reducing physiological thermal indices. After calving, (i.e., postpartum, during the subsequent lactation) active cooling was provided to both groups, and DMI (16.8 vs. 18.0 ± 0.8 kg/day, *p* = 0.32), RR, and RT (*p* > 0.43) were similar between treatment groups. 

**Figure 1:**
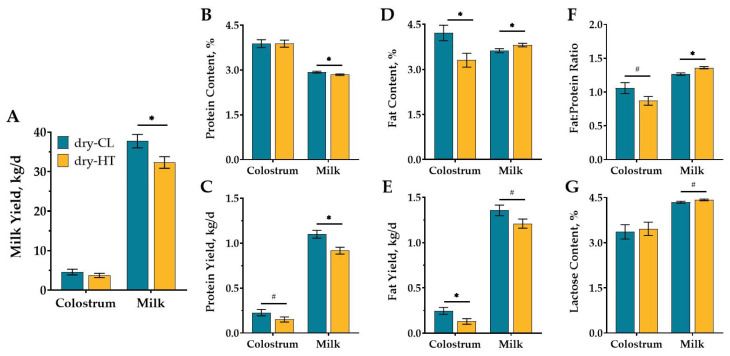
Effect of dry period heat stress on milk yield and composition. Multiparous Holstein cows were exposed to dry period heat stress (dry-HT, shade only, *n* = 12) or cooling (dry-CL, shade, fans, and water soakers, *n* = 12) for 46 days before calving. After calving, all cows were actively cooled and colostrum and milk yield (**A**) and composition (i.e., protein (**B**,**C**), fat (**D**,**E**), fat:protein ratio (**F**) and lactose (**G**)) were measured daily and averaged weekly in AfiFarm up to 210 DIM. Composition is reported as content (%), ratio, or yield (kg/day; calculated from milk yield). All data are presented as least square means ± standard error of the treatment. * indicates *p* ≤ 0.05; # indicates 0.05 < *p* ≤ 0.10.

Dry-HT cows yielded 5.4 kg/day less milk compared to dry-CL cows across 210 DIM (*p* = 0.03; Figure 1A), but colostrum yield did not significantly differ (3.7 vs. 4.6 ± 0.6 kg; *p* = 0.36). Milk yield differed over time (*p* (DIM) < 0.01), but there was no interaction between treatment and week in milk (*p* (TRT × DIM) = 0.34, Supplementary Figure S2). Colostrum did not significantly differ in protein content between treatment groups (Figure 1B). Dry-HT cows had decreased colostrum protein yield by 0.08 kg, but this difference was a tendency (*p* = 0.10; Figure 1C). Dry-HT cows experienced a 0.09% overall reduction in milk protein content and a 0.18 kg/d loss in protein yield across 210 DIM compared to dry-CL, despite provision of active cooling to both groups during lactation (*p* < 0.04; Figure 1B,C). Milk protein content and yield varied across 210 DIM for both groups, but there was no significant interaction between treatment and time (Figure S2). Colostrum fat content and yield were decreased by 0.9% and 0.12 kg in dry-HT cows compared to dry-CL cows (*p* < 0.03; Figure 1D,E). Conversely milk fat content was higher in dry-HT cows (*p* = 0.05; Figure 1D), though milk fat yield tended to be reduced compared to dry-CL cows (*p* = 0.08; Figure 1E). Similar to fat content outcomes, colostrum fat: protein ratio tended to be reduced in dry-HT cows (*p* = 0.08; Figure 1F), but the milk fat: protein ratio was significantly greater for dry-HT cows compared with dry-CL cows (*p* = 0.002; Figure 1F). Colostrum lactose content did not significantly differ, and milk lactose content only tended to increase in dry-HT cows relative to dry-CL cows (*p* = 0.08; Figure 1G).

### 3.2. Milk Protein Profile and Plasma and Tissue AA 

**Figure 2:**
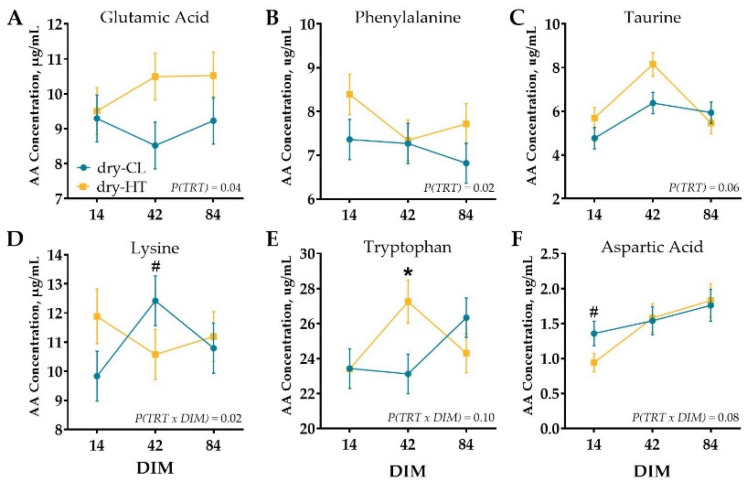
Plasma free AA concentration at 14, 42, and 84 days in milk (DIM). Cows were exposed to dry period heat stress (dry-HT, *n* = 5) or cooling (dry-CL, *n* = 5) for 46 days pre-calving. Blood was collected at 14, 42, and 84 DIM, and plasma was analyzed for free AA concentration. Amino acids that differed between treatments (TRT, **A**–**C**) or treatment × DIM interaction (**D**–**F**) are depicted with significant contrasts denoted by * (*p* ≤ 0.05) or # (0.05 < *p* ≤ 0.10). All data are presented as least square means ± standard error of the treatment × time interaction.

Milk protein fractions did not significantly differ between treatment groups, and there was no treatment by DIM interaction (*p* > 0.16; Table 1). Similarly, there were no statistical differences in mammary tissue AA concentration (*p* > 0.18; Supplementary Figure S3). Venous AA profile differed slightly between treatments, but of the 36 free AA and metabolites measured, 15 did not differ signficantly over DIM, treatment, or interaction (*p* > 0.10) and 15 were changed over time only (*p* (DIM) ≤ 0.10; Supplementary Table S2). However, dry-HT cows had higher overall concentrations of glutamic acid and phenylalanine (*p* ≤ 0.04) and tended to have higher taurine concentrations (*p* = 0.06; Table S2, Figure 2A–C) during lactation relative to dry-CL cows. There was a treatment by DIM interaction for lysine whereby dry-HT cows had lower concentrations of lysine at 42 DIM relative to dry-CL cows (*p* (TRT × DIM) = 0.02; Table S2, Figure 2D). There were tendencies for an interaction for tryptophan and aspartic acid; whereby dry-HT cows had elevated tryptophan concentrations at 42 DIM but lower aspartic acid concentrations at 14 DIM (*p* (TRT × DIM) ≤ 0.10; Table S2, Figure 2E,F). 

### 3.3. Mammary Tissue AA Transporter Gene Expression

**Figure 3:**
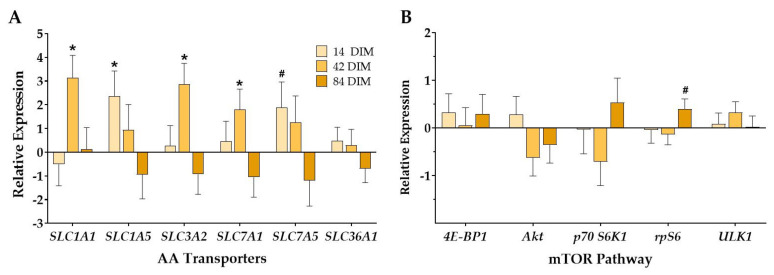
Effect of dry period heat stress on mammary tissue amino acid transporter (**A**) and mTOR pathway (**B**) gene expression. Cows were exposed to dry period heat stress (dry-HT, *n* = 6) or cooling (dry-CL, *n* = 6) for 46 days pre-calving. Mammary biopsies were collected at 14, 42, and 84 days in milk (DIM), and RNA was extracted from mammary tissue to analyze gene expression via qRT-PCR. Gene expression is reported as relative expression of dry-HT cows relative to dry-CL cows. * indicates *p* ≤ 0.05; # indicates 0.05 < *p* ≤ 0.10.

All mammary AA transporters evaluated herein, except for *SLC36A1*, had a treatment by DIM interaction whereby dry-HT cows had upregulated expression of AA transporters at either 14 or 42 DIM only (*p* (TRT × DIM) ≤ 0.03; Figure 3A, Supplementary Table S3). The treatment by DIM interaction was a stastitical tendency for *SLC1A5* and *SLC7A5* (*p* (TRT × DIM) ≤ 0.08). More specifically, dry-HT cows had increased mRNA expression of *SLC1A1*, *SLC3A2*, and *SLC7A1* at 42 DIM relative to dry-CL cows (*p* ≤ 0.05; Figure 3A). The transporters *SLC1A5* and *SLC7A5* were significantly upregulated and tended to be upregulated, respectively, at 14 DIM in dry-HT cows relative to dry-CL cows (*p* = 0.04 and *p* = 0.10; Figure 3A). There was also a DIM effect for *SLC1A5*, *3A2*, *7A1*, and *7A5* (Supplementary Table S3). 

### 3.4. Mammary Tissue mTOR Gene and Protein Expression

There were few differences between treatments in mammary expression of mTOR pathway genes. There were no significant effects for treatment, but there was a treatment by DIM interaction for *rpS6* whereby dry-HT cows showed upregulated expression of *rpS6* at 84 DIM (*p* (TRT × DIM) = 0.04; Figure 3B). All genes had a significant effect of day (*p* < 0.01). 

**Figure 4:**
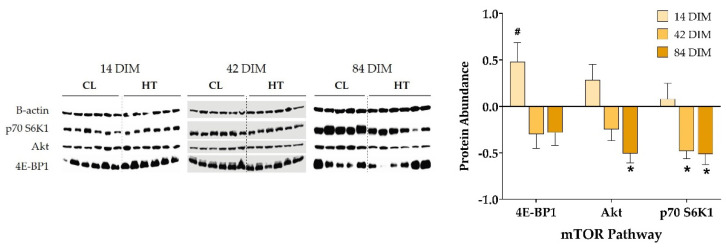
mTOR pathway protein abundance. Cows were exposed to dry period heat stress (dry-HT, *n* = 6) or cooling (dry-CL, *n* = 5) for 46 days pre-calving. Mammary biopsies collected at 14, 42, and 84 days in milk (DIM) were used for protein extraction to analyze mTOR pathway protein abundance by western blotting (Original picture, please see File S1). Protein expression is reported as abundance of dry-HT cows relative to dry-CL cows, normalized to β-actin. All data are presented as least square means ± standard error. * indicates *p* ≤ 0.05; # indicates 0.05 < *p* ≤ 0.10.

In contrast with gene expression results, dry period heat stress increased mammary protein abundance of 4E-BP1 at 14 DIM (*p* = 0.02; Figure 4). In addition, dry period heat stress significantly reduced mammary protein abundance of p70 S6K1 at 42 DIM and of p70 S6K1 and Akt at 84 DIM (*p* ≤ 0.05; Figure 4). A visual depiction of the relative expression of AA and mTOR pathway genes (i.e., ΔCt) and proteins (i.e., abundance) for each treatment group can be found in Supplementary Figure S4.

## 4. Discussion

Hyperthermia during lactation impairs both milk production and milk protein synthesis due to direct and indirect alterations to a number of biological systems [5,9,10]. While extensive research demonstrates decreased milk production due to dry period heat stress, there is conflicting evidence on its effect on milk protein synthesis and composition in the subsequent lactation [22,23,24]. The current study explores the impact of dry period heat stress on milk protein content and determines the carry-over impact on mechanistic pathways involved in milk protein synthesis during lactation, specifically AA availability and mTOR signaling. 

During the dry period, RR and RT were elevated in the cows exposed to heat stress relative to those provided heat stress abatement despite similar THI, indicating successful heat stress abatement for the dry-CL group, consistent with previous studies [22,23]. Notably, there was no significant difference in dry period DMI between treatment groups, which is dissimilar to past studies where dry period heat-stressed cows consumed on average 13% less than their dry period cooled counterparts [20,38]. Upon calving, all cows were provided heat abatement across the duration of the lactation. The dry period CL and HT cows had similar DMI and comparable RR and RT within thermoneutral ranges as lactating cows, suggesting that the heat stress abatement during lactation was successful in both groups and neither group was experiencing a significant heat load [39]. This lack of difference in postpartum DMI, concurrent with the subsequent lactation, is consistent with previous studies of dry period (i.e., prepartum) heat-stressed versus cooled dams [23,38,40]. Thus, we suggest that alterations during lactation related to nutrient partitioning and signaling can be partially attributed to heat stress exposure during the dry period.

After calving, dry period heat-stressed cows produced 5.6 kg/day less milk. This lower production is within the range of previous findings, though the above literature review averages; these report that dams heat-stressed during late-gestation produced 3 to 7.5 kg/d less milk compared with dams provided access to shade, fans, and soakers, even when both groups were cooled during lactation [20,22,23]. While the colostrum protein content did not differ between treatments, colostrum protein yield and milk protein content and yield were significantly decreased in cows exposed to dry period heat stress. This decline in milk protein concentration could be costly to dairy producers, as most U.S. milk marketing orders employ a multiple component pricing system that compensates producers based on milk protein, as well as fat and other solids. Indeed, Bailey et al. found that if milk protein concentration fell one standard deviation below the mean, milk value would be reduced by approximately 7% or $0.82/cwt [41]. Analysis of economic losses due to dry period heat stress has yet to take into account any impact on milk composition [21], thus warranting further modeling and investigation to more accurately predict financial losses. 

Interestingly, while milk protein content was significantly decreased during lactation after dry period heat stress exposure, lactose content did not differ, and fat content and fat: protein ratio surprisingly increased; however, fat yield was decreased as a consequence of reduced milk yield. The increase in milk fat concentration and fat: protein ratio is inconsistent with previous studies in heat-stressed lactating and dry cows that found either no difference or decreased milk fat concentrations in heat-stressed groups [8,11,22,23,42]. Indeed, an RNA-Seq analysis of mammary metabolism of mid-lactation dairy cows under heat stress found overall downregulation of genes and pathways related to mammary tissue lipid metabolism [43]. Thus, the nature of our results necessitates further research, such as assessing dilution effects or differentiating between de novo versus pre-synthesized fatty acids, which is beyond the scope of the current analysis. 

The current study did not find any difference in milk protein fractions, while past research in lactating dairy cows reports alterations in milk protein profile upon direct exposure to high temperatures or across different seasons. In particular, studies show an overall lower casein concentration, a decrease in the proportion of αs2-casein and increase in the proportion of αs1-casein, or a reduction in αs-casein and β-casein concentrations under heat stress or in the summer months [11,18]. The phosphorylation of α- and β-caseins requires ATP [44], thus these authors postulated that the reduced energy and protein availability under heat stress may contribute to the alterations in protein fraction [18]. Dry period heat stress does not have a significant impact on DMI during lactation with little-to-moderate impact on postpartum NEFA, BHBA, and glucose concentrations [40,45,46]. Interestingly, a study assessing seasonal heat stress during late gestation found elevated NEFA and reduced plasma insulin and glucose during lactation; however, the cattle were unable to maintain thermoneutrality postpartum despite the provision of active cooling, which may have influenced metabolic outcomes [46]. Herein, the carry-over impact of dry period heat stress may not have influenced metabolic energy availability to a degree that would alter milk protein fraction as seen in heat stress during lactation. 

In the present study, the lack of difference in postpartum DMI during lactation lends credence to the idea that additional physiological and molecular systems may be regulating milk protein metabolism after exposure to prepartum heat stress. However, the numerical reduction in prepartum DMI during dry period heat stress or more nuanced alterations in energy partitioning not found in the present study could also contribute to this. Another possible contributor is reduced availability of AA and metabolites for milk protein synthesis. In the current study, systemic plasma Glu, Phe, and taurine had overall increased concentrations in dry period heat stressed cows across early and peak lactation. Previous studies in lactating cows show both increases [47] and decreases [9,10,43] in AA availability in hyperthermic conditions. Consistent with the current results, Guo and others found an increase in the concentration of blood Glu but a decrease in Lys concentration in heat stressed lactating cows [47]. Glutamic acid supports the local immune function and is required for heat-shock protein structural and functional integrity [48,49,50]. Although not directly studied in bovine heat stress studies, Phe supplementation in vitro increased the expression of *HSP70* mRNA in bovine renal epithelial cells [51], and Phe metabolism assists in the suppression of T-cell immune responses [52]. Similarly, taurine supplementation in chronically heat-stressed poultry has been shown to promote heat shock protein expression and enhance protein synthesis [53,54]. Thus, upregulation of these AAs in circulation might play a role in assisting in the increased immune and heat shock responses associated with heat stress, both during the dry period and during lactation [43,55]. 

Further, our results show interaction effects; for instance, plasma Lys, a limiting essential AA, tended to decrease specifically at 42 DIM (i.e., roughly peak milk production) in dry-HT cows compared with dry-CL cows. Lysine is one of the most limiting AA for milk protein synthesis [56], and Lys stimulates milk protein synthesis, partly by promotion of amino acid transporter B (0+) and activation of the mTOR pathway in bMEC [57]. Though, the plasma AA measured herein represents the systemic AA profile and thus caution should be taken when inferring its specific impacts on mammary metabolism. Notably, there is considerable interest in infusing limiting AA to improve milk and protein yields [58], but additional infusion of Lys (as well as Met and branched-chain AA) has been shown to alter milk protein content with no impact on milk or protein yields during heat stress in lactating cows [59]. Thus, supplementing dry cows with the essential AA may aid in modulating milk protein content but might not be an effective tool to combat the impact of dry period heat stress on milk yield. Further research should test this experimentally. 

The amino acid transporter genes *SLC7A5* and *SLC1A5* had increased expression at 14 DIM in dams heat-stressed in late gestation relative to cooled dams. Later at 42 DIM, transporter genes *SLC1A1*, *SLC3A2*, and *SLC7A1* were also upregulated in dry period heat-stressed cows. A comprehensive study by Bionaz and Loor [33] demonstrates that the transporter genes measured herein are responsible for encoding a variety of proteins that are responsible for active pumping of AA into mammary tissue (*SLC1A1* and *1A5*) or the counter-transport of cationic AA (*SLC3A2*, *7A1*, *7A5*). Of the transporters upregulated around peak lactation (i.e., 42 DIM) in the current study, *SLC1A1* encodes EAAT3 for the transport of AA such as Glu. The gene *SLC3A2* assists in guiding LAT1 to the cell membrane though synthesis of heavy polypeptide chain 4f2hc, leading to the transport of essential AA like Phe and Met. The protein CAT1 is encoded by *SLC7A1* and transports Lys and Arg. We suggest that this orchestrated upregulation of genes encoding for AA transporters in dry-HT cows at early and peak lactation may be a compensatory mechanism of the mammary gland to increase mammary AA availability and consequently drive milk protein synthesis and cellular growth. Kaufman et al. [16] measured the same set of AA transporter genes in a bMEC in vitro direct heat stress model. They also found an upregulation in the gene expression of *SLC1A1* and *SLC3A2* after 12 h of 41.5 °C heat exposure and proposed a similar mechanism–increased AA transport activity could be a cellular adjustment to maintain AA uptake, protein synthesis, and bMEC mass [16,60]. Interpretation relative to the present study requires caution, however, as the dairy cows herein were exposed to heat stress during their dry period and not to direct hyperthermia during lactation. 

Along with an increase in AA transporter gene expression, Kaufman and collaborators reported a decrease in phosphorylation of the insulin transductor and mTORC1 activator Akt and of the mTORC1 downstream substrate rpS6 [16]. Phosphorylation of Akt was also reduced in skeletal muscle of gilts upon short term heat stress [61]. In the present study, dry period heat stress reduced Akt protein abundance at 84 DIM. It also reduced protein abundance of mTORC1 substrate and rpS6 kinase p70 S6K1 at 42 and 84 DIM. Intriguingly, mTORC1 inhibition stimulates the expression of the mRNA translational repressor 4E-BP1, which stimulates mRNA expression of AA transporter genes *SLC3A2*, *SLC7A1*, and *SLC7A5* [62], potentially as a coping mechanism of AA deficiency. Herein, dry period heat stress tended to increase 4E-BP1 abundance at 14 DIM, confirming previous in vitro results at the mRNA level [63]. Finally, though not measured in the present study, systemic circulating insulin could have played a role in milk protein metabolism via the mTORC1 pathway. Insulin activates mTORC1 signaling and mTORC1 kinase activity, and in turn, mTORC1 controls insulin signaling and sensitivity to modulate biological functions such as cell division and growth and protein translation [64]. While dry period heat stress does not influence prepartum insulin metabolism, it has been shown to promote elevated circulating insulin and glucose in early lactation [45]. 

Finally, it should be noted that the limited sample size in the present study (*n* = 6 per treatment for molecular analysis and *n* = 12 per treatment for physiological measures and milk and component yields) could contribute to the lack of significant differences observed in dry matter intake and milk protein profiles. Thus, results and further application should be interpreted with caution. 

## 5. Conclusions

Our results indicate an orchestrated response of the lactating mammary gland to prior dry period heat stress exposure, in part mediated by the mTORC1 pathway. This includes the upregulation of AA transporter gene expression to possibly overcome AA deficiency, along with a repression of milk protein production, which extends from early lactation to mid-lactation. Factors affecting plasma or mammary tissue AA concentrations during lactation in response to dry period heat stress require additional investigation. More research in this area is necessary to ensure production of high-quality and -quantity milk as a sustainable source of protein in the face of rising global temperatures.

## Figures and Tables

**Table 1:** Effect of dry period heat stress on milk protein profile during lactation. Cows were exposed to dry period heat stress (dry-HT, *n* = 6) or cooling (dry-CL, *n* = 6) for 46 days pre-calving. Milk was collected at 14, 42, and 84 days in milk (DIM) and analyzed for milk protein fractions. Data are presented as least square means ± standard error of the treatment (TRT).

Protein (%)	Treatment			*p*-Value		
	Dry-CL	Dry-HT	SEM	TRT	DIM	TRT × DIM
Low Molecular Weight	13.42	13.38	1.06	0.95	<0.01	0.52
Peptides	0.51	0.77	0.12	0.19	0.02	0.96
α-Lactalbumin	3.84	3.73	0.23	0.84	0.77	0.43
β-Lactoglobulin	8.04	8.96	0.59	0.74	0.25	0.59
α-S1 Casein	31.76	32.14	0.61	0.48	0.08	0.56
α-S2 Casein	7.28	7.64	0.45	0.81	0.18	0.88
β-Casein	31.30	29.69	0.85	0.20	0.13	0.74
γ-Casein	0.57	0.68	0.10	0.30	0.13	0.49
κ-Casein	3.38	3.17	0.19	0.37	0.73	0.16

## Data Availability

All data is available within this manuscript.

## Supplementary Materials

The following are available online at https://www.mdpi.com/article/10.3390/ani11092676/s1, Figure S1. Effect of dry period heat stress on milk yield and composition over time, Figure S2. Effect of dry period heat stress on milk yield and composition over time, Figure S3. Mammary tissue amino acid (AA) concentrations, Figure S4. Effect of dry period heat stress on relative expression of mammary AA transporter genes, mTOR pathway genes, and mTOR pathway proteins during lactation. Table S1. Primer sequences for validated and housekeeping genes, Table S2. Effect of dry period heat stress on plasma free amino acid (AA) concentration during lactation, Table S3. Effect of dry period heat stress on relative expression of mammary AA transporter genes during lactation. File S1: original western blotting picture.
