# Supplementary material for: Dry Period Heat Stress Impacts Mammary Protein Metabolism in the Subsequent Lactation
Source: Animals (Basel). 2021 Sep 13;11(9):2676. doi: 10.3390/ani11092676 (PMC8466034; doi:10.3390/ani11092676)
Supplement: Supplementary file 1 [file animals-11-02676-s001.zip › animals-1323378-supplementary/animals-1323378-SM.pdf]

Dry period heat stress impacts mammary protein metabolism in the subsequent lactation

Bethany Dado-Senn[1], Amy L. Skibiel[2], Geoffrey E. Dahl[2], Sebastian I. Arriola Apelo[1], and

Jimena Laporta[1,*]

Table S1. Primer sequences for validated and housekeeping genes.

Gene Symbol	Accession Number	5′ ->3′	Primer Sequence	Source
SLC1A1	U72534	F. 880	GGCTTGCAATCCACTCCATT	[31]
		R. 980	CAGAAGAGCCTGGGCCATT	
SLC1A5	BC123803	F. 850	CTGGTGAAGGTGCCCACTG	[31]
		R. 978	GAAGAAGCGAATGAGCAGCTC	
SLC3A2	BC102420	F. 861	GTGTGGACGGGTTTCAGGTC	[31]
		R. 961	CCGATCCTCACTGACGCTCT	
SLC7A1	DQ399522	F. 418	CTTCGACCTGAAGGACCTGG	[31]
		R. 518	GCTCGGGCTGGTATCGTAAG	
SLC7A5	BC126651	F. 753	GGGTGACGTAGCCAATCTGG	[31]
		R. 859	ATCCCCCATAGGCAAAGAGG	
SLC36A1	CB438466	F. 39	ACCTGCCCAACTGTTGGCT	[31]
		R. 140	TGATGATCTCGGCAGGGAC	
4E-BP1	BC120290	F: 357	TTTGAGATGGACATTTAAAGGGC	[31]
		R: 457	CTTGCATAAGGCCTGGCTG	
rpS6	NM_001015548	F: 150	CTGGGTGAAGAATGGAAGGG	[31]
		R: 250	CGAACTCTGCCATGGGTCA	
p70 S6K1	DN544771	F: 250	CAAGCTTGCATGCTAATTTGTCC	[31]
		R: 350	TTGAGTCCTGATCATGTCGAAGA	
Akt	NM_173986	F: 1565	CACGTGCTCTGGACGCTTC	[31]
		R: 1666	ATGGCGAGGTTCCACTCAAAC	
ULK1	NC_037344.1	F: 68	CAGAACTACCAGCGCATTGA	[58]
		R: 176	GTGCAAAGCCCAAAGGACT	
RPS9	NM_001101152	F: 192	GGAGACCCTTCGAGAAGTCC	[30,32]
		R: 254	CTTTCTCATCCAGCGTCAGC	
UXT	XM_004022128.3	F: 323	TGTGGCCCTTGGATATGGTT	[32]
		R: 423	GGTTGTCGCTGAGCTCTGTG	

[58] Untergasser, A.; Cutcutache, I.; Koressaar, T.; Ye, J.; Faircloth, B.C.; Remm, M.; et al. Primer3-new capabilities and interfaces. *Nucleic Acids Res.* **2012**, *40*, 1–12. doi:10.1093/nar/gks596

Figure S1. qRT-PCR melting peaks of amino acid (AA) and mTOR pathway genes assessed.

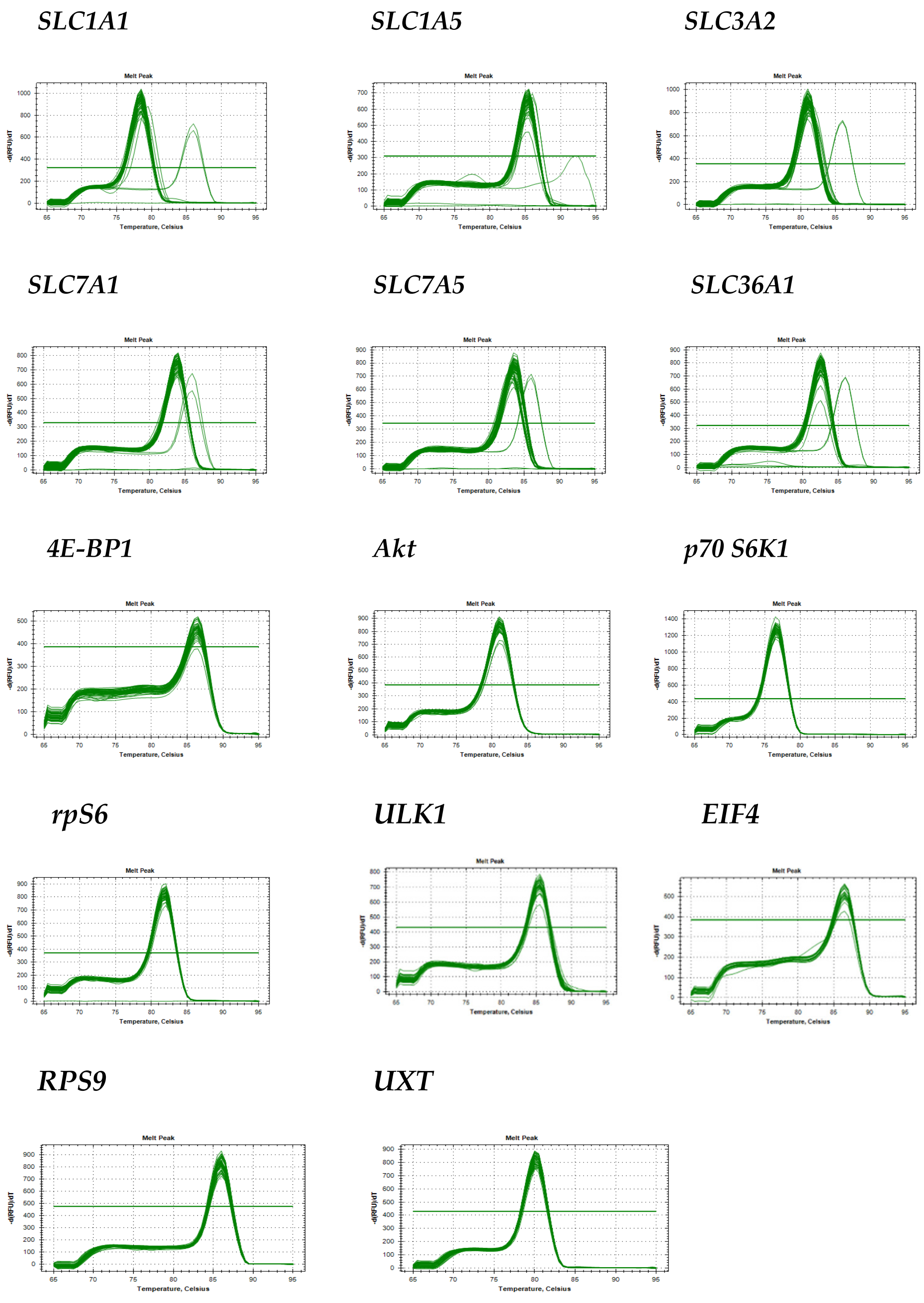

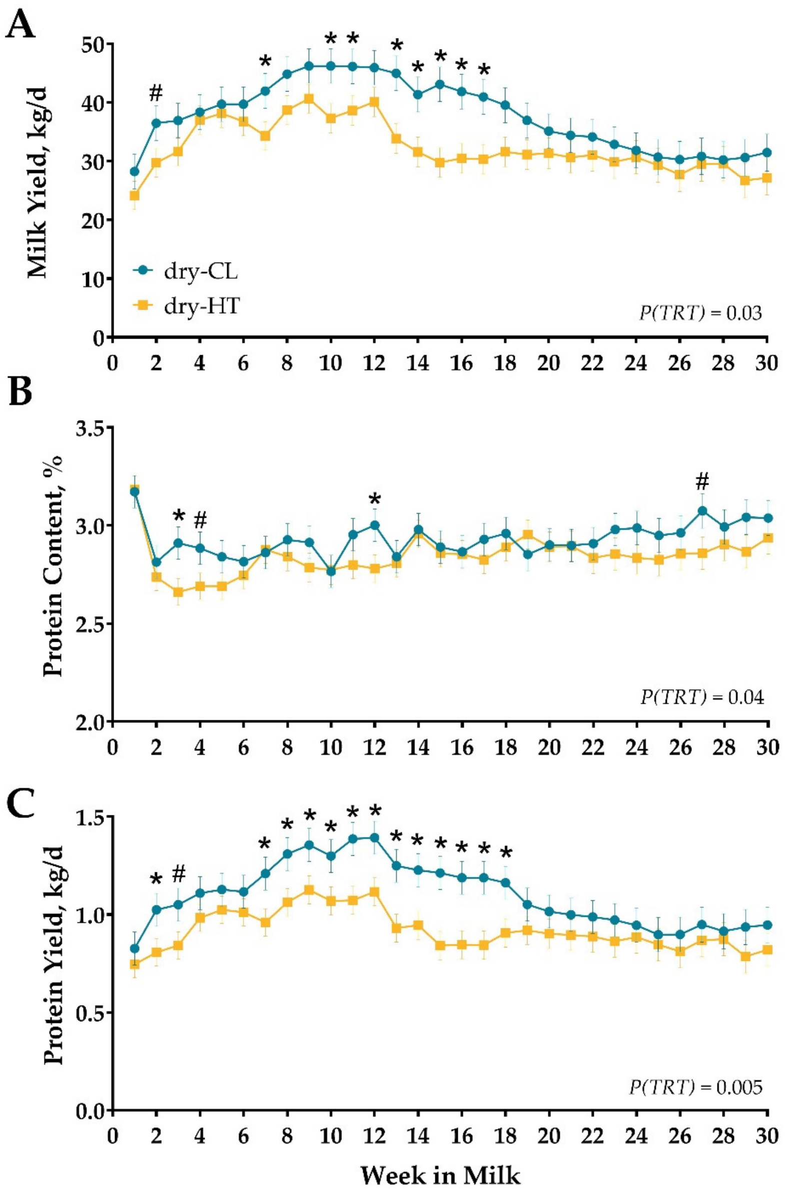

Figure S2. Effect of dry period heat stress on milk yield and composition over time. Multiparous Holstein cows were exposed to dry period heat stress (dry-HT, shade only, n = 12) or cooling (dry-CL, shade, fans, and water soakers, n = 12) for 46 d before calving. After calving, all cows were actively cooled and colostrum and milk yield and protein were measured daily and averaged weekly in AfiFarm up to 210 DIM. Milk protein is reported as content (%) and yield (kg/d; calculated from milk yield). All data are presented as LSM ± SE of the treatment x week in milk interaction. * indicates $P \leq 0.05$; # indicates $0.05 < P \leq 0.10$.

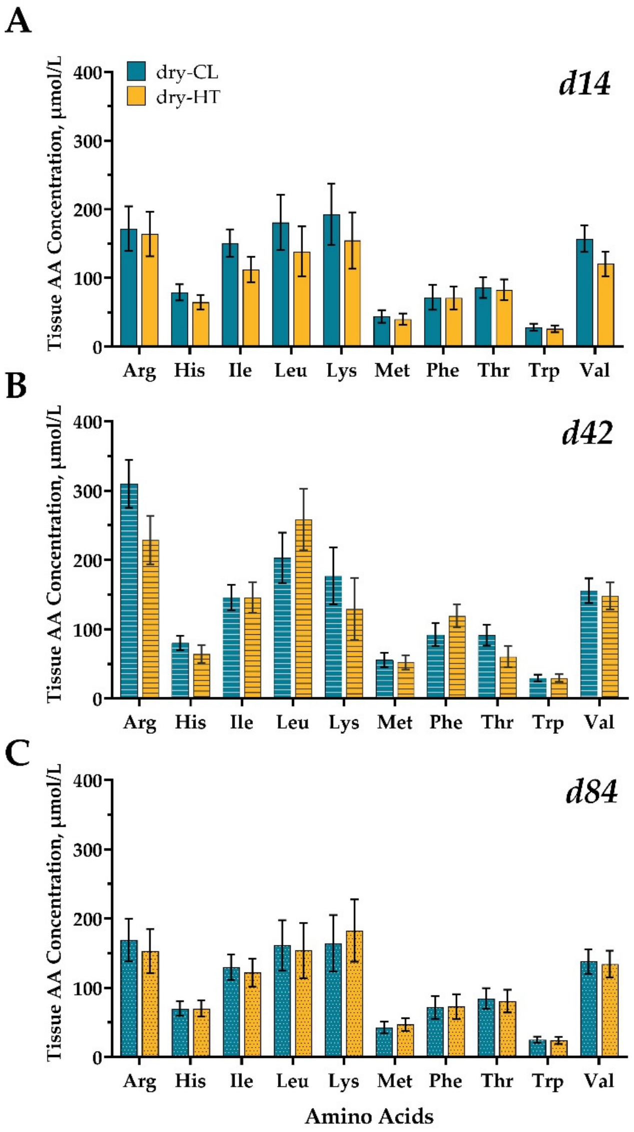

Figure S3. Mammary tissue amino acid (AA) concentrations. Multiparous Holstein cows were exposed to dry period heat stress (dry-HT, shade only, n = 12) or cooling (dry-CL, shade, fans, and water soakers, n = 12) for 46 d before calving. After calving, both groups had access to shade, fans, and water soakers. Mammary biopsies collected at 14, 42, and 84 DIM were used to analyze AA concentration via LCMS. All data are presented as LSM ± SE of the treatment at each time point.

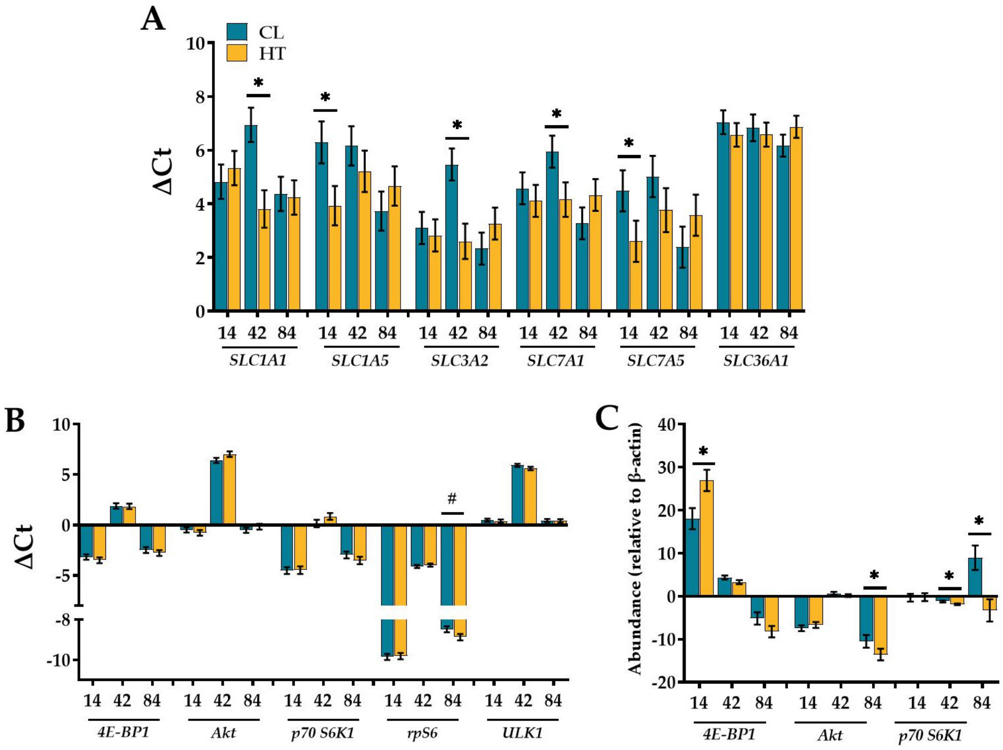

Figure S4. Effect of dry period heat stress on relative expression of (**A**) mammary AA transporter genes, (**B**) mTOR pathway genes, and (**C**) mTOR pathway proteins during lactation. Cows were exposed to dry period heat stress (dry-HT, n = 6) or cooling (dry-CL, n = 6) for ~46 d pre-calving. Mammary biopsies were collected at 14, 42, and 84 DIM (after treatment exposure), and RNA and protein were extracted from mammary tissue for analysis. Gene expression (**A**,**B**) is reported as the ΔCt, the relative expression compared to housekeeping genes. A higher ΔCt indicates a lower expression relative to the housekeeping genes. Protein abundance (**C**) is relative to the protein β-actin. Data are presented as LSM ± SE.

Table S2. Effect of dry period heat stress on plasma free amino acid (AA) concentration during lactation. Cows were exposed to dry period heat stress (dry-HT, n = 5) or cooling (dry-CL, n = 5) for 46 d pre-calving. Blood was collected at 14, 42, and 84 DIM and plasma was analyzed for free AA concentration. Data are presented as LSM ± SE of the treatment.

	TRT			*p*-value		
Amino acid (μg/mL)	dry-CL	dry-HT	SEM	TRT	DIM	TRT×DIM
α-amino adipic acid	1.64	1.50	0.26	0.70	0.29	0.71
α-amino n-butyric acid	1.56	1.59	0.08	0.79	0.08	0.23
Alanine	20.60	19.53	1.64	0.66	0.72	0.40
Arginine	12.00	12.26	0.80	0.82	0.01	0.51
Asparagine	4.11	4.04	0.31	0.88	0.09	0.29
Aspartic Acid	1.54	1.40	0.16	0.53	<0.01	0.08
Carnosine	6.07	6.67	0.40	0.32	<0.01	0.45
Citrulline	14.59	14.60	1.04	0.99	0.03	0.17
Cystathionine	0.41	0.40	0.03	0.94	0.32	0.83
Ethanolamine	0.22	0.19	0.02	0.43	0.02	0.97
Glutamic acid	9.02	10.17	0.34	0.04	0.76	0.48
Glutamine	29.55	26.33	2.03	0.30	0.34	0.45
Glycine	23.28	24.81	2.40	0.67	<0.01	0.54
Histidine	7.07	6.78	0.48	0.69	0.98	0.84
Homocystine	0.55	0.67	0.10	0.41	0.34	0.21
Hydroxylysine	0.06	0.05	0.02	0.70	0.58	0.92
Hydroxyproline	1.77	1.96	0.16	0.43	0.17	0.79
I methyl histidine	2.62	2.67	0.20	0.88	0.02	0.37
III methyl histidine	0.65	0.61	0.07	0.72	<0.01	0.83
Isoleucine	13.36	13.35	0.63	0.99	0.58	0.88
Leucine	19.68	18.86	1.21	0.65	0.66	0.82
Lysine	11.02	11.22	0.65	0.83	0.56	0.03
Methionine	3.10	3.14	0.16	0.89	0.02	0.78
Ornithine	5.61	5.78	0.37	0.75	0.06	0.98
Phenylalanine	7.13	8.01	0.21	0.02	0.36	0.89
Phosphoserine	1.19	1.09	0.06	0.22	0.06	0.15
Proline	10.84	11.75	0.75	0.42	0.89	0.55
Sarcosine	1.23	1.18	0.10	0.75	0.47	0.37
Serine	10.09	10.07	0.41	0.97	0.01	0.68
Taurine	5.70	6.44	0.24	0.06	0.01	0.14
Threonine	10.22	10.33	0.60	0.90	0.77	0.19
Tryptophan	24.29	24.99	0.52	0.38	0.19	0.10
Tyrosine	7.04	7.94	0.39	0.14	0.05	0.23
Urea	232.89	221.79	14.33	0.60	0.05	0.56
Valine	27.79	26.67	1.76	0.66	0.23	0.85
λ-amino butyric acid	0.09	0.08	0.04	0.85	0.65	0.22

Table S3. Effect of dry period heat stress on relative expression of mammary AA transporter genes during lactation. Cows were exposed to dry period heat stress (dry-HT, *n* = 6) or cooling (dry-CL, *n* = 6) for ~46 d pre-calving. Mammary biopsies were collected at 14, 42, and 84 DIM (after treatment exposure), and RNA was extracted from mammary tissue to analyze amino acid transporter gene expression. Gene expression is reported as the ΔCt, the relative expression compared to housekeeping genes. Data are presented as LSM± SE.

Gene of Interest	TRT			*P*-value		
(relative expression)	dry-CL	dry-HT	SEM	TRT	DIM	TRT*DIM
SLC1A1	5.38	4.46	0.41	0.14	0.23	0.02
SLC1A5	5.39	4.60	0.57	0.35	0.04	0.06
SLC3A2	3.63	2.90	0.38	0.20	0.08	0.01
SLC7A1	4.60	4.20	0.43	0.53	0.05	0.03
SLC7A5	3.96	3.32	0.57	0.44	0.08	0.08
SLC36A1	6.68	6.66	0.23	0.96	0.78	0.34
