# Supplementary figures and images for: Dry Period Heat Stress Impacts Mammary Protein Metabolism in the Subsequent Lactation
Source: Animals (Basel). 2021 Sep 13;11(9):2676. doi: 10.3390/ani11092676 (PMC8466034; doi:10.3390/ani11092676)

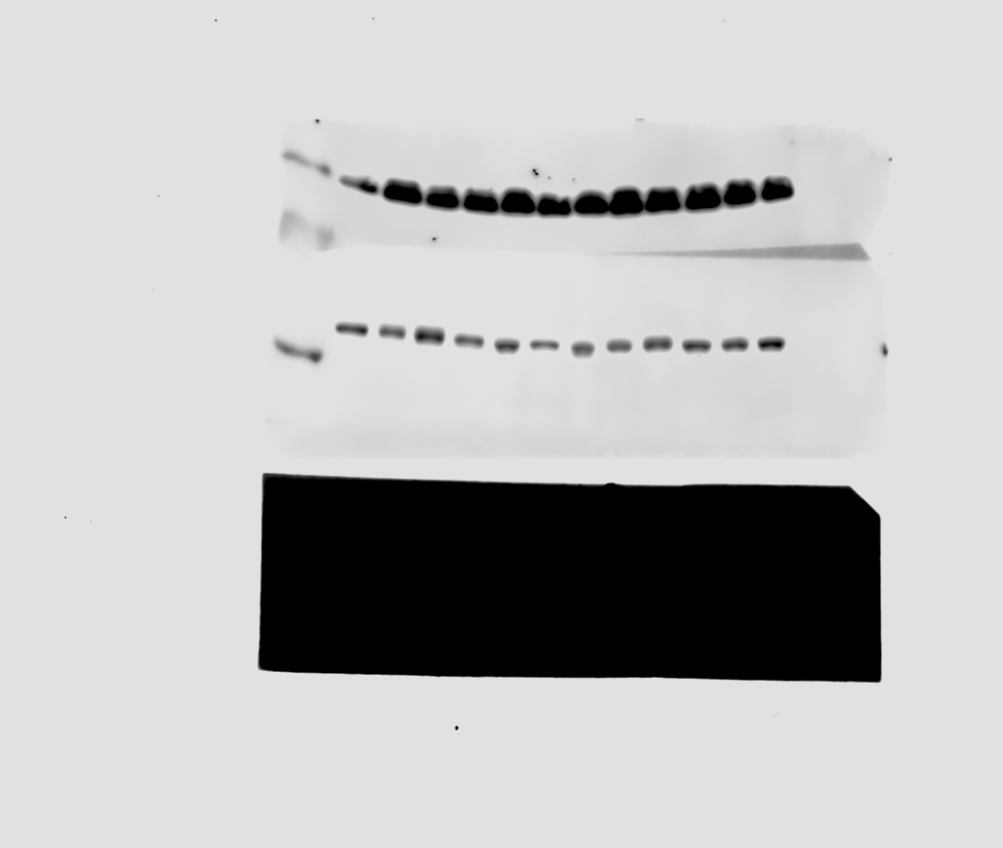

Supplement: Supplementary file 1 [file animals-11-02676-s001.zip › animals-1323378-supplementary/File S1-Original Western Blot figure/0000025_01 14DIM 4EBP1 top strip S6K1 bottom.tif]

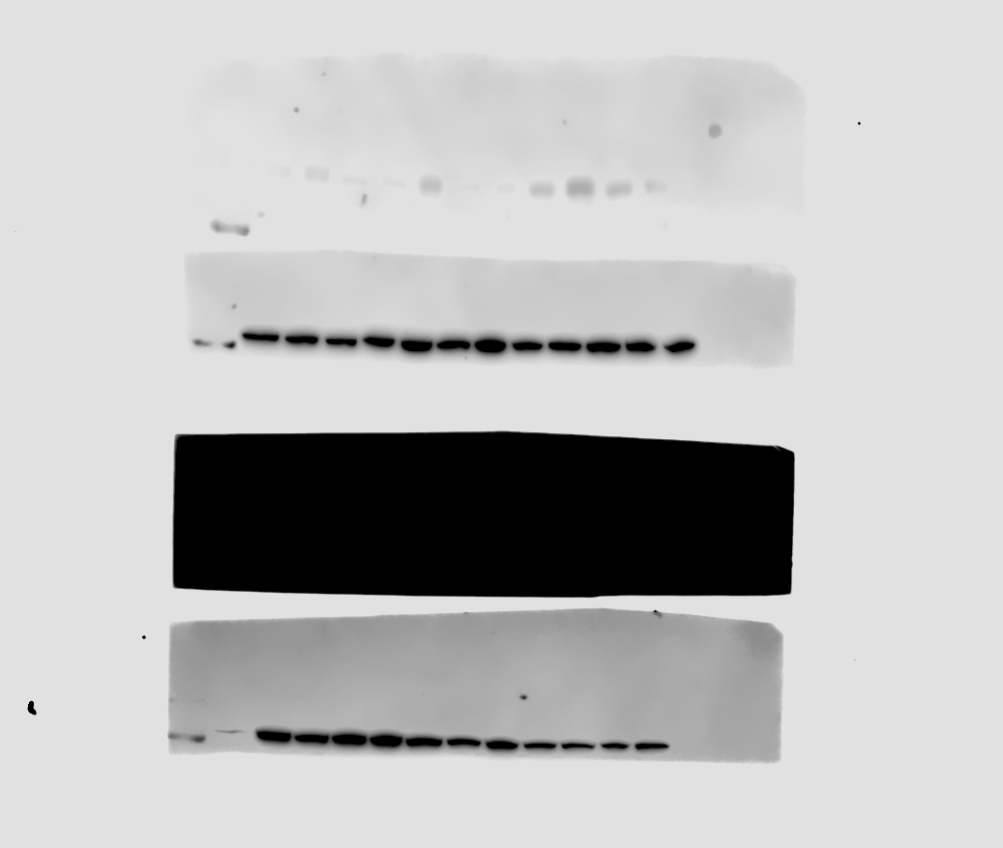

Supplement: Supplementary file 1 [file animals-11-02676-s001.zip › animals-1323378-supplementary/File S1-Original Western Blot figure/0000026_01 AKT 14DIM 2ndTop AKT 84DIM bottom.tif]

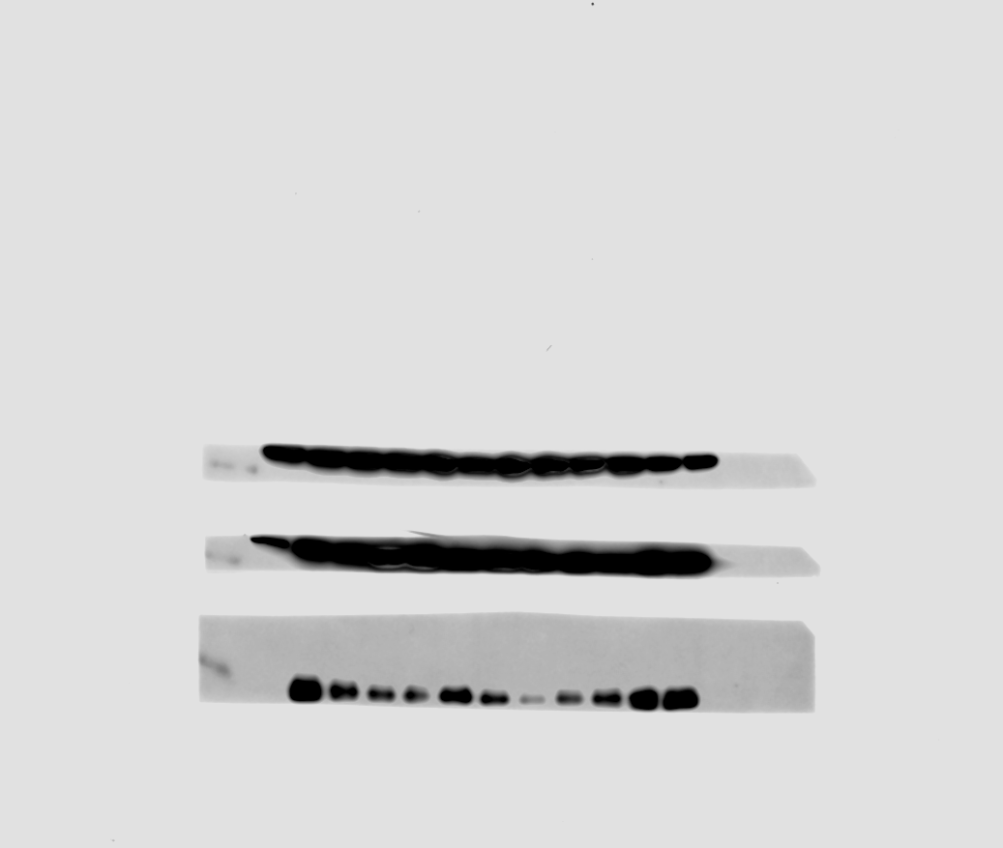

Supplement: Supplementary file 1 [file animals-11-02676-s001.zip › animals-1323378-supplementary/File S1-Original Western Blot figure/0000030_01 84DIM 4EBP1 bottom.tif]

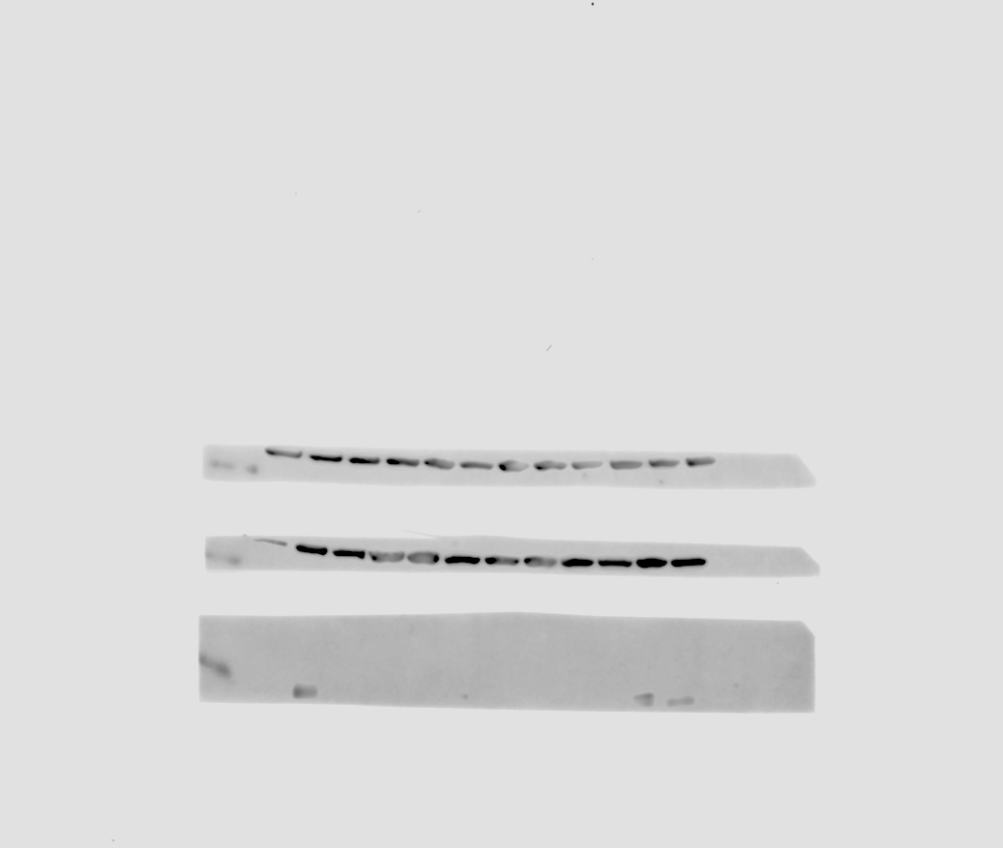

Supplement: Supplementary file 1 [file animals-11-02676-s001.zip › animals-1323378-supplementary/File S1-Original Western Blot figure/0000030_01 Actin 14DIM top 84DIM middle.tif]

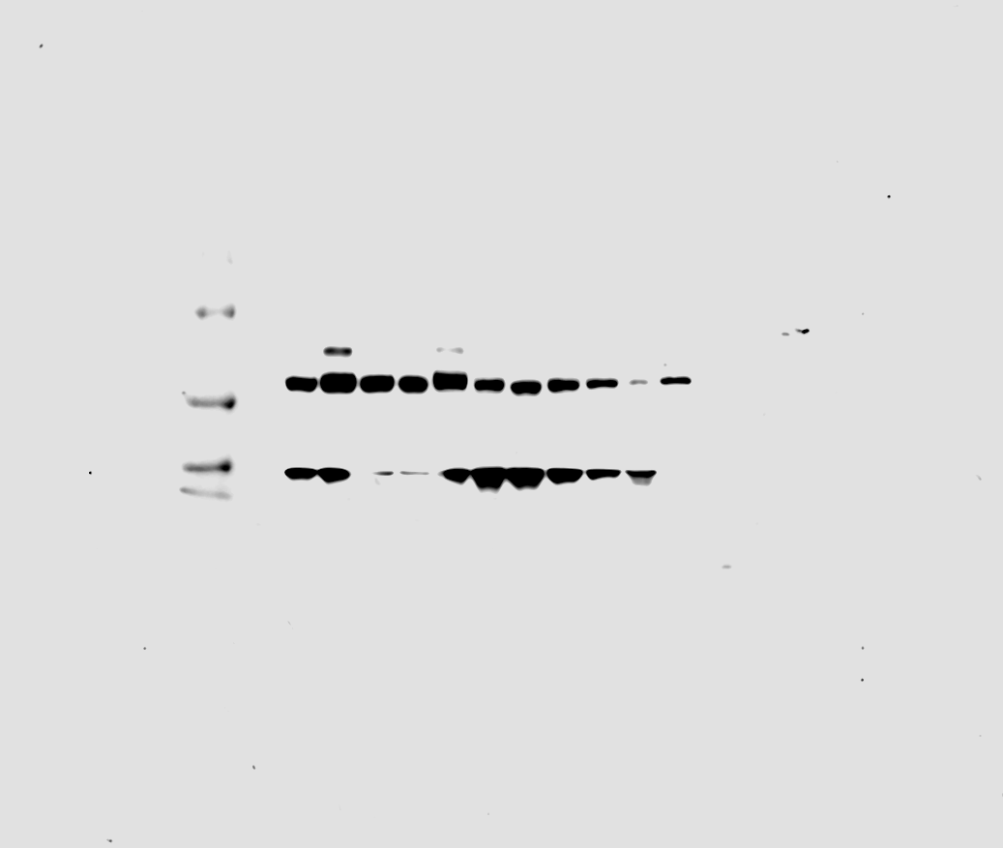

Supplement: Supplementary file 1 [file animals-11-02676-s001.zip › animals-1323378-supplementary/File S1-Original Western Blot figure/0000031_01 84DIM S6K1 Top band.tif]

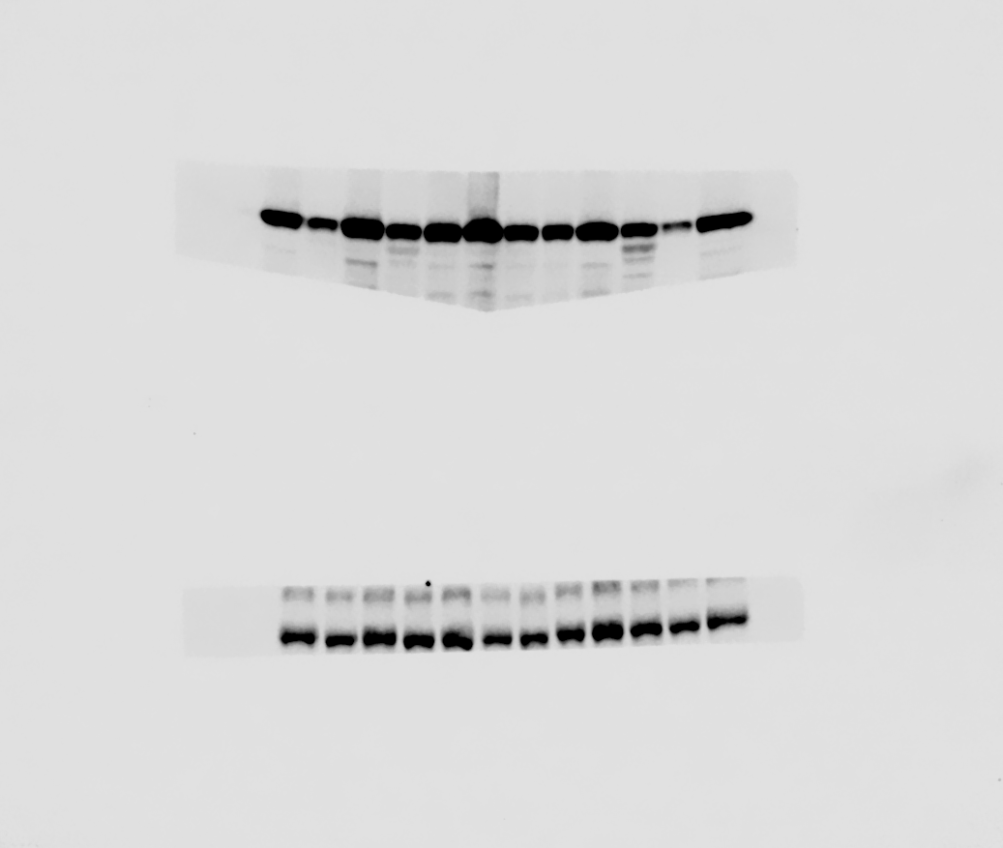

Supplement: Supplementary file 1 [file animals-11-02676-s001.zip › animals-1323378-supplementary/File S1-Original Western Blot figure/0000107_01 42DIM S6K1 Bottom strip.tif]

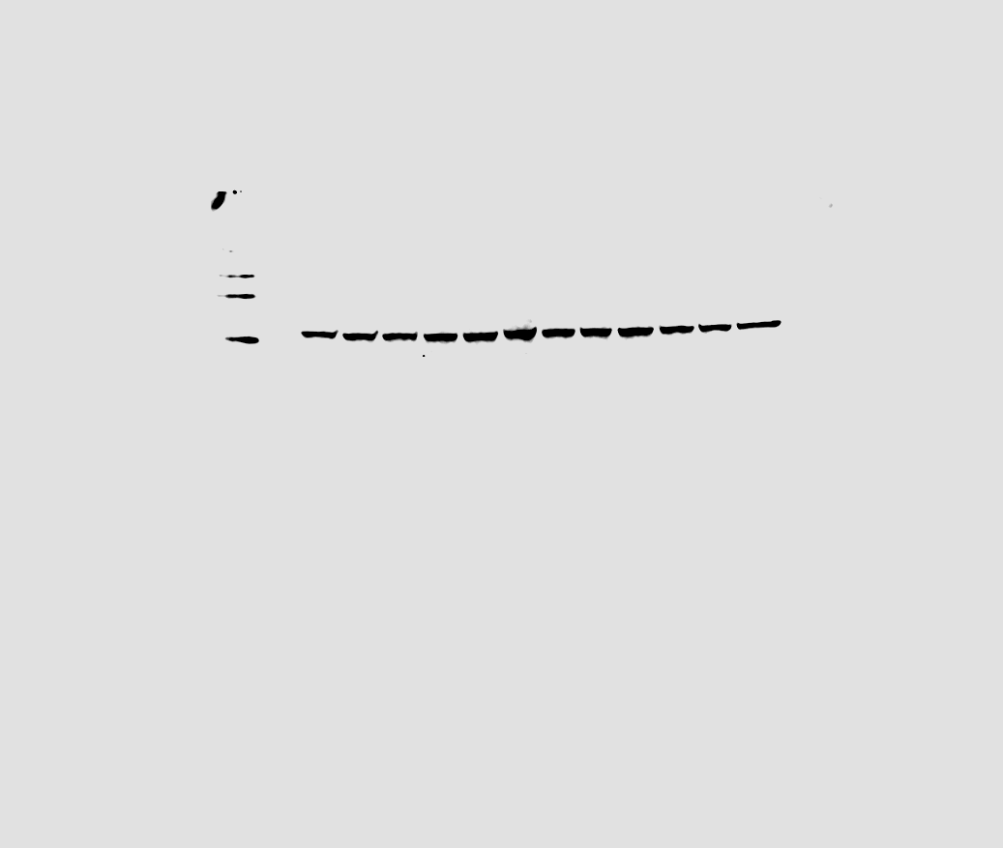

Supplement: Supplementary file 1 [file animals-11-02676-s001.zip › animals-1323378-supplementary/File S1-Original Western Blot figure/0000108_01 42DIM Akt.tif]

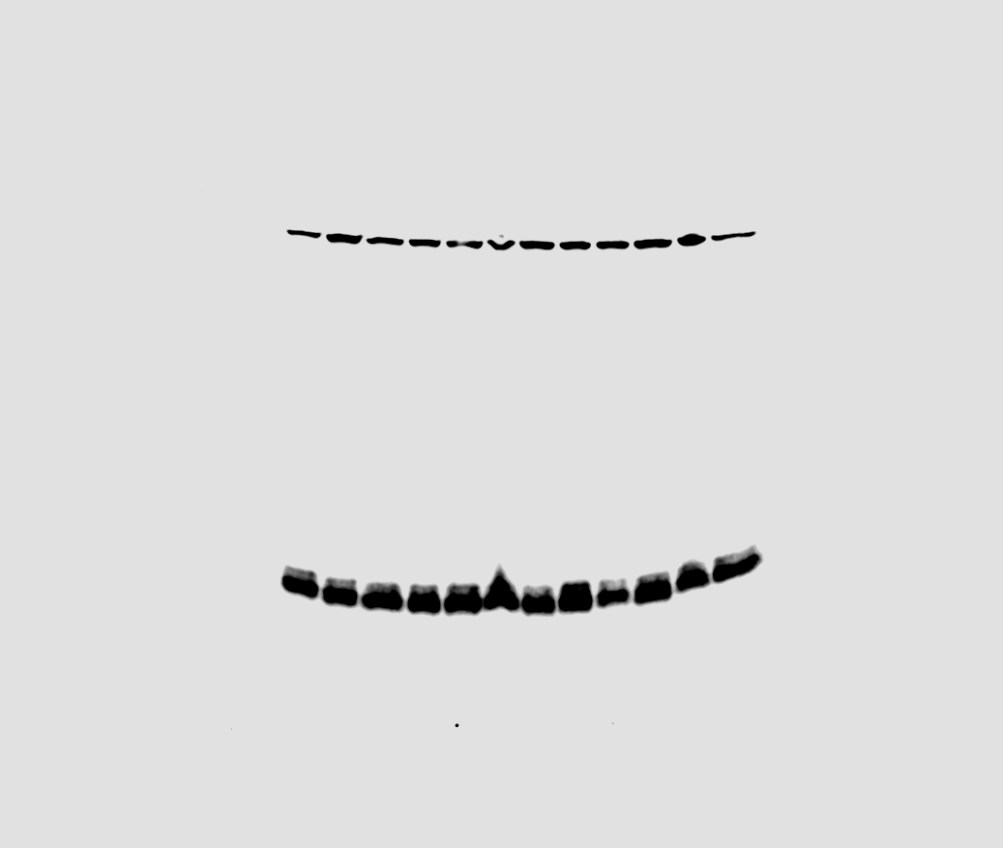

Supplement: Supplementary file 1 [file animals-11-02676-s001.zip › animals-1323378-supplementary/File S1-Original Western Blot figure/0000159_01 42DIM Actin top 4EBP1 bottom strip without 700 channel.tif]

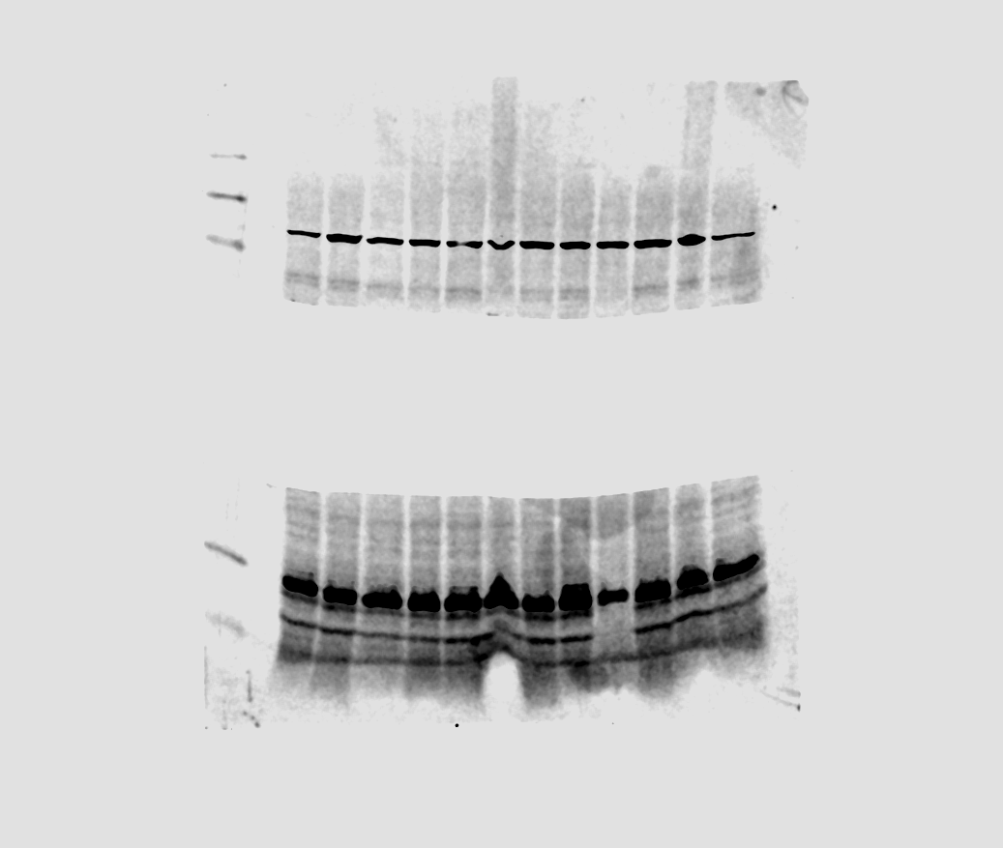

Supplement: Supplementary file 1 [file animals-11-02676-s001.zip › animals-1323378-supplementary/File S1-Original Western Blot figure/0000159_01 42DIM Actin top 4EBP1 bottom strip.tif]
